# The Beneficial Effect of Acute Exercise on Motor Memory Consolidation is Modulated by Dopaminergic Gene Profile

**DOI:** 10.3390/jcm8050578

**Published:** 2019-04-27

**Authors:** Lasse Christiansen, Richard Thomas, Mikkel M. Beck, Jessica Pingel, Jeppe D. Andersen, Cameron S. Mang, Mads A. J. Madsen, Marc Roig, Jesper Lundbye-Jensen

**Affiliations:** 1 Department of Nutrition, Exercise and Sports, University of Copenhagen, 2200 Copenhagen, Denmark; mib@nexs.ku.dk (M.M.B.); madsjm@drcmr.dk (M.A.J.M.); jlundbye@nexs.ku.dk (J.L.-J.); 2 Department of Neuroscience, University of Copenhagen, 2200 Copenhagen, Denmark; jessica.pingel@gmail.com; 3 Copenhagen Centre for Team Sport and Health, University of Copenhagen, 2200 Copenhagen, Denmark; 4 Department of Forensic Medicine, University of Copenhagen, 2200 Copenhagen, Denmark; jeppe.dyrberg.andersen@sund.ku.dk; 5 Faculty of Kinesiology and Health Studies, University of Regina, Regina, SA S4S 0A2, Canada; Cameron.Mang@uregina.ca; 6 School of Physical and Occupational Therapy, McGill University, Montreal, QC H3G 1Y5, Canada; marc.roigpull@mcgill.ca; 7 Memory and Motor Rehabilitation Laboratory (MEMORY-LAB), Feil and Oberfield Research Centre, Jewish Rehabilitation Hospital, Montreal Centre for Interdisciplinary Research in Rehabilitation (CRIR), Laval, QC H7M 3L9, Canada

**Keywords:** physical activity, consolidation, dopamine, genetics, motor learning, single-nucleotide polymorphisms, dopamine receptor

## Abstract

When aerobic exercise is performed following skilled motor practice, it can enhance motor memory consolidation. Previous studies have suggested that dopamine may play a role in motor memory consolidation, but whether it is involved in the exercise effects on consolidation is unknown. Hence, we aimed to investigate the influence of dopaminergic pathways on the exercise-induced modulation of motor memory consolidation. We compared the effect of acute exercise on motor memory consolidation between the genotypes that are known to affect dopaminergic transmission and learning. By combining cluster analyses and fitting linear models with and without included polymorphisms, we provide preliminary evidence that exercise benefits the carriers of alleles that are associated with low synaptic dopamine content. In line with previous reports, our findings implicate dopamine as a modulator of the exercise-induced effects on motor memory consolidation, and suggest exercise as a potential clinical tool to counteract low endogenous dopamine bioavailability. Further experiments are needed to establish causal relations.

## 1. Introduction

Successful memory formation depends on both encoding and the subsequent consolidation of memory traces [1]. Cardiovascular exercise has been demonstrated as an endogenous neuromodulator with the potential to benefit both procedural and declarative learning and memory by facilitating encoding and consolidation processes [2,3,4,5]. However, the learning-enhancing effect of acute exercise is associated with substantial individual differences potentially arising from genetic variation in neurophysiological signaling systems [6]. Elucidating the impact of functional gene variants provides insight into the neurophysiological mechanisms underlying exercise-induced improvements in memory consolidation [7], and further qualifies the use of individualized exercise interventions as a tool to improve motor learning (e.g., neurorehabilitation) [8]. In this short communication, we report results from an exploratory retrospective investigation of the neurogenetic basis for individual differences in the effect of exercise on memory consolidation with a particular emphasis on dopaminergic neurotransmission. 

The possibility of using acute aerobic exercise to enhance memory encoding has been investigated extensively (see e.g., [9] for a review). In contrast, experiments targeting consolidation through exercise after memory encoding are only just beginning to emerge. Within the declarative memory domain, results have been equivocal, which may reflect differences between the employed memory tasks along with differences in exercise timing and intensity [10]. In contrast, exercise is consistently reported to benefit motor memory consolidation [11,12,13]. We have previously reported that aerobic exercise performed early (<1 h) and later (~2 h) during the consolidation phase (i.e., after motor practice) positively affects long-term retention of motor skills [14,15,16,17]. The mechanisms underlying the effects of exercise on memory consolidation are poorly understood. Conceptually, memory enhancement can be achieved by potentiating and/or stabilizing the encoded engram, but also by protecting it against interfering influences [18] (see also Beck et al., in preparation). The neural circuitries affected by exercise and the involved mechanisms are sparsely studied, but could include alterations in cortical, subcortical, and corticospinal transmission [19,20]. 

### Dopamine Transmission Influences Memory Consolidation

Exercise conducted prior to memory encoding has been demonstrated to benefit motor memory formation through mechanisms involving catecholaminergic and neurotrophic activity [21,22]. Both systemic and central nervous concentrations in dopamine (DA) increase with exercise [23,24,25,26,27,28,29], also suggesting that DA-related mechanisms are likely to contribute to the observed behavioral effects of exercise. Early findings of the strain-dependent effects of dopaminergic agents on memory consolidation in mice [30] lend credence to the tenet that genetic variations may moderate the effect of exogenous (e.g., L-dopa [31]) and endogenous (e.g., exercise [32]) modulators of the dopaminergic system. In support of this, Mang et al. (2017) recently demonstrated that a single nucleotide polymorphism (SNP) in *ANKK*, which is known to influence the central nervous expression of the dopamine D2 (*DRD2*) receptor, predicted the effect acute of exercise upon motor learning in humans [32]. However, importantly, exercise was performed prior to motor practice. This may influence both acquisition and later retention e.g., by increasing neuropsychological phenotypes such as arousal, and the effect can thus not be ascribed to the consolidation processes per se. In contrast, exercise conducted after encoding (i.e., post-trial exercise) benefits consolidation through direct neurochemical actions. Post-trial DA manipulation modulates the consolidation of both declarative [33,34,35,36] and procedural [37] memory. Here, we extend the findings from Mang et al. (2017) and explore the interactions between the genetic variations that are known to influence dopaminergic signaling and aerobic exercise performed post-motor practice on motor skill consolidation and long-term motor memory retention. 

## 2. Study Design and Data Analysis

**Figure 1:**
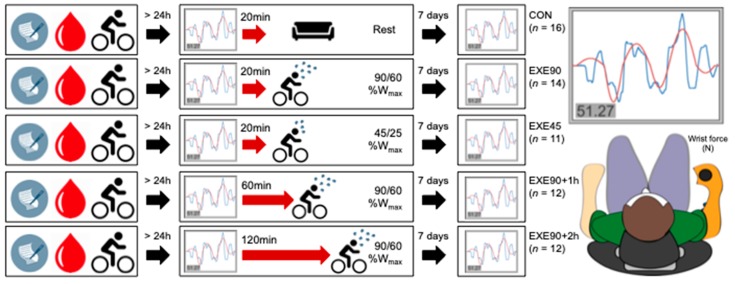
Experimental Design and Visuomotor Accuracy Task (VAT). (**Left**) A schematic illustration of the study design. Participants reported to the laboratory four times. The first visit encompassed questionnaires, blood sampling, and a graded exercise test. The main experiment (second visit, min. 24 h after the first visit) included motor skill acquisition and subsequent exercise or rest intervention. Delayed retention tests were conducted one and seven days after motor skill practice. The one-day retention test is not depicted here, and was not considered in the current analyses. (**Right**) The behavioral set-up for motor skill acquisition and retention. Participants were seated in a comfortable chair in front of a monitor with their right, dominant arm strapped in a customized carbon fiber half-cast grasping a fixed handle with a built-in force transducer. By applying wrist flexion or extension force, participants could trace the displayed target (red) as accurately as possible, informed by the augmented numeric visual feedback.

We investigated the influence of genetic variation in DNA purified from whole blood samples collected between 2013–2015 from participants enrolled in two previously reported studies [14,15] and 13 participants in a preceding pilot project. It was not possible to obtain blood samples in eight of 60 participants from the previous reports. Accordingly, blood samples were genotyped for 65 able-bodied male participants. The experimental paradigm is outlined in Figure 1. All the participants practiced a visuomotor accuracy task (VAT) involving isometric wrist flexion and extension force production as described in previous reports [14,15] before engaging in either aerobic exercise or a passive control protocol. Following standardized introduction and familiarization to the task, participants practiced the VAT in five training blocks of 20 trials each. Baseline motor performance was defined as the mean score in block one, and block five represented the post-acquisition motor performance. 

Participants were allocated to the intervention groups depicted in Figure 1, and these groups were matched for cardiovascular fitness, age, and baseline motor performance. Following motor practice, the control group rested, whereas participants in the other groups performed exercise at either intense or moderate intensity immediately after acquisition, one hour later, or two hours later (see Table A1 in Appendix A for participant characteristics). 

Long-term retention of the encoded memory was tested one and seven days later by a single block of 20 trials with no augmented feedback, and the mean score was used as a measure of retention. Memory consolidation was operationalized by computing the change in performance from the final block of motor practice to the retention test seven days later. 

DNA was extracted from anticoagulated whole blood using the QIAamp DNA Mini Kit (Qiagen, Hilden, Germany). Genotyping was performed using the iPLEX^®^ Gold kit (Agena Bioscience, Inc., Hamburg, Germany). The samples were spotted in duplicates using the RS1000 Nanospotter (Agena Bioscience, Inc., Hamburg, Germany) and visualized on the MassARRAY^®^ analyzer 4 system (Agena Bioscience, Inc., Hamburg, Germany) using the autorun settings. Samples were analyzed with a Typer Analyzer 4 (Agena Bioscience, Inc., Hamburg, Germany). As argued by Frank and Fossella (2010), an inherent drawback of a candidate gene design is the risk of complex interaction with other loci, which may render the effects of a single allele on a continuous dependable variable undetectable in small heterogeneous populations [38]. This warrants a hypothesis-driven approach that allows some exploration. We analyzed eight SNPs and one variable number of tandem repeats (VNTR) in eight genes previously demonstrated to influence motor learning through their impact on the central nervous bioavailability of dopamine and brain-derived neurotrophic factor (BDNF). These are presented in Table 1. Experimental procedures and data analysis including the behavioral model, description of participants, genotyping, and data reduction are described in Appendix A. 

## 3. Results and Discussions

### 3.1. Variations in DRD2, PPP1R1B, and SLC6A3 Influence the Effects of Exercise

Four loci on four genes all involved in dopamine transmission (*DRD2*: rs1076560, *ANKK1*: rs180049, PPP1R1B/*DARPP-32*: rs907094, and *DAT1/SLC6A3*: rs28363170) were found to either interact with exercise or to have an isolated effect on the consolidation score, and were subsequently included in a descriptive linear mixed effect model (see Table 1). 

We evaluated the variance accounted for by the models by means of marginal and conditional R-squared values and compared measures of goodness of fit in models including exercise and all four SNPs by means of the Akaike Information Criteria (AIC) (see Table 2) from linear mixed models. A similar approach has previously been applied to elucidate the interactive effects of dopaminergic genotypes and L-dopa on motor skill learning [31]. 

**Figure 2:**
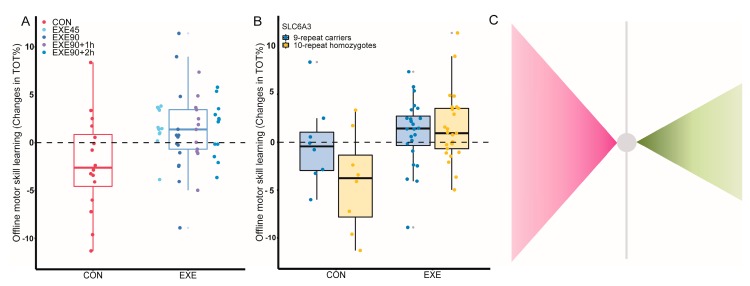
The effect of exercise and gene variance on consolidation. (**A**) The effect of exercise on motor skill consolidation with rest condition (red) on the left and the four different exercise protocols depicted in different shades of blue. Note that data analysis is conducted with the four groups collapsed to one. The boxes range from the first to the third quartile with second quartile (median) depicted as the horizontal line. Whiskers represent 1.5 times the interquartile range between the first and third quartile. The scatter plots represent individual data points, and outliers are marked with an adjacent grey dot. (**B**) The effect of the variable number of tandem repeats (VNTR) polymorphism in the SL6A3 gene. Note the ‘recover’ effect of exercise for 10 repeat carriers. (**C**) Papillion-plot illustrating the variance explained by the identified SNPs. Vertical ranges of the triangles depict the variation of off-line changes i.e., the inversed relative proportion of variance in relation to the full model explained by the model with (pink) and without SNPs in the model. Note how the capacity to explain the variance in the effects of exercise on skill retention changes when SNPs are introduced in the model, suggesting an interaction. Values are derived from model estimates.

The four identified SNPs relating to dopaminergic transmission explained a substantial part of the variance in the exercise-induced enhancements of motor memory consolidation. As such, the influence of exercise alone accounted for 10% (R^2^(m) = 0.10) of the variance in motor memory consolidation, whereas the complete model including exercise and the four identified SNPs accounted for 19% (R^2^(m)) = 0.19) of this variance (illustrated in Figure 2C). Furthermore, corrected planned comparisons revealed statistically significant effects of exercise for carriers of gene variants associated with low endogenous DA availability (see below). The limitation of the between-subject design and the uneven allele distribution and sample sizes warrant caution when ignoring the influence from the remaining SNPs. Nevertheless, the results support our hypothesis that aerobic exercise affects motor memory consolidation, in part, through dopamine-dependent processes. 

The involvement of dopaminergic neurotransmission in motor learning is well-supported in the literature. Dopamine plays a crucial role in reinforcement learning involving reward prediction errors [39] leading to stronger motor memories through improved consolidation [40,41]. Also, long-term motor learning has been demonstrated to be DA-dependent [42,43,44]. At a synaptic level, dopamine has meta-plastic effects, setting the threshold for synaptic modification [45]. Structural changes such as synaptogenesis is also affected through DA-mediated increases in cortical [46] and striatal [47,48] expression of the immediate early gene c-fos. Thus, dopamine stimulates structural changes that are necessary for long-term memory [49]. 

### 3.2. Exercise Benefits Individuals with Allele Combinations Associated with Lower Consolidation

**Figure A1:**
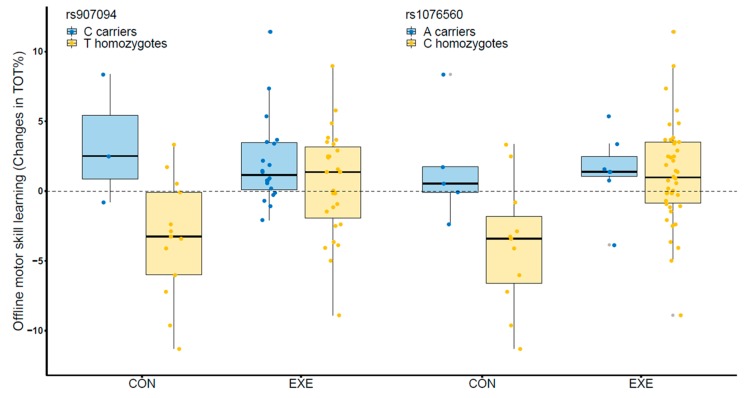
The effects of exercise and gene variations in *PPP1R1B (locus: rs907094)* and *DRD2 (locus: rs1076560)* on motor memory consolidation. The boxes range from the first to the third quartile, with the second quartile (median) depicted as the horizontal line. Whiskers represent 1.5 times the interquartile range between the first and third quartile. The scatters represent individual data points, and outliers are marked with an adjacent grey dot.

The intronic SNP, rs1076560 (C > A) in *DRD2*, has previously been demonstrated to influence neural activity in motor-related cortical and subcortical areas [50,51]. The polymorphism has been reported to affect the expression of both pre and post-synaptic DRD2 receptors [52]. The minor A allele is associated with less presynaptic DRD2 autoinhibition in the striatum, and consequently more synaptic DA activity [53]. In addition, we further genotyped a VNTR at the rs28363170 locus of *DAT1/SLC6A3* to investigate the potential influence of decreased dopamine transporter (DAT) expression and resultant higher synaptic DA associated with the 9-repeat allele [54]. We found that homozygotic C at the rs1076560 locus in *DRD2*, as well as homozygotic 10 repeats individuals in the *DAT1/SL6A3*, displayed higher performance when exercise took place after motor practice exercise (*DRD2* EXE_C_-CON_C_: 5.10 ± 1.29, *p* < 0.001; DAT1/SLC6A3 EXE_10_-CON_10_: 5.62 ± 1.58, *p* = 0.002) (Figure 2B and Figure A1). In agreement with the extant literature, we found carriers of T and 9 repeats to display a marginally higher degree of off-line skill improvement within the resting control group (DRD2 CON_A_–CON_C_: 5.51 ± 2.05, *p* = 0.03; DAT1/SLC6A3 CON_9_–CON_10_: 3.92 ± 1.94, *p* = 0.14) (Figure 2B and Figure A1). Collectively, this indicates that exercise can be speculated to act as a putative endogenous intervention strategy to counteract the detrimental effects of low dopamine bioavailability on motor skill consolidation. This speculation remains to be substantiated by future experiments. 

The rs907094 locus in *PPP1R1B*, encoding the dopamine-regulated and cAMP-regulated phosphoprotein of molecular weight 32 kDa (DARPP-32), was shown to relate to motor skill consolidation independently and in interaction with exercise. DARPP-32 is highly expressed in dopaminoceptive areas such as the neostriatum [55,56], with the highest expression in individuals with rs907094:A [57]. The intricate regulation of DARPP-32 makes predictions of effects difficult, but it has been suggested as a potent regulator of synaptic strength and plasticity [58] through the DRD1-mediated inhibitory control of protein phosphatase 1 [59] (but see also [60,61] for a review). In T homozygotes, we found larger off-line learning effects in individuals exercising after motor practice as compared to resting (*PPP1R1B* EXE_T_-CON_T_: 3.97 ± 1.27, *p* = 0.006), which counteracted the lower motor skill consolidation observed in T homozygotes not exercising compared to C carriers (PPP1R1B CON_T_-CON_C_: −6.79 ± 2.41, *p* = 0.02) (Figure A1); however, the small population of resting control C carriers impedes conclusive comparisons. 

### 3.3. Val66Met Polymorphism Did Not Influence Consolidation or Interact with Exercise

The effect of the *ANKK1* glu713lys (Taq1A, glutamic acid to lysine) substitution did not reach conventional standards for statistical significance for the interaction with exercise and time (*p* = 0.09). Nevertheless, our findings suggesting that glu/glu participants benefitted more from exercise as compared to lys carriers dovetail with earlier findings by Mang et al. (2017). 

Subsequently, we genotyped the Val66Met polymorphism (*BDNF*) to enable statistical corrections in case of a main effect or an interaction with exercise. This SNP results in a valine to methionine substitution on position 66, and has been demonstrated to influence plasticity in the motor system and the effect of exercise on memory [62,63,64]. In agreement with Mang et al., we did not find this SNP to mediate the effect of exercise, and did not correct the model based on the BDNF SNP [32]. Additionally, the val158met polymorphism in *COMT* (rs4680), encoding the catechol-O-methyltransferase enzyme highly expressed in prefrontal cortex, did not interact with exercise or influence consolidation independent of exercise. A similar finding using a comparable behavioral model has been reported previously [31]. However, our null findings should be interpreted cautiously due to the unbalanced allele frequency. 

## 4. Conclusions

In summary, our results imply a role for the SNPs that have been previously demonstrated to impact synaptic dopamine levels along with the striatal expression of plasticity-regulating proteins in modulating the effect of exercise on motor memory consolidation. We suggest future research to establish causal relations by blocking or enhancing dopamine transmission during and following aerobic exercise. The current dataset has inherit limitations due to its small sample size, the between-subject design, and the different exercise protocols. Nevertheless, our findings provide important, albeit preliminary results implicating dopaminergic signaling pathways in mediating the beneficial effects of exercise on motor memory consolidation. The present data add to the existing literature suggesting a role for aerobic exercise in patients characterized by dopamine scarcity by attenuating neurochemical deficits [65,66]. 

## Figures and Tables

**Table 1:** Effects of ‘SNP’ main effect, ‘Time × SNP’ and ‘Exercise × Time × SNP’ interactions extracted from linear mixed effect model (LMM) analyses. *p*-values are computed using Satterthwaite’s method employed in the *lmerTest* R-package. SNP: single nucleotide polymorphism. Bold numbers denote significant main effects and interactions.

Gene	Locus	~ SNP	~ Time × SNP	~ Exercise × Time × SNP
***DRD2***	rs1076560	0.16	**0.03**	**0.04**
***DRD2***	rs6277	0.18	0.36	0.77
***ANKK1***	rs1800497	0.26	**0.02**	0.09
***DRD1***	rs686	0.09	0.15	0.63
***DRD3***	rs6280	0.12	0.48	0.74
***COMT***	rs4680	0.71	0.44	0.42
***PPP1R1B***	rs907094	0.56	**0.003**	**0.05**
***SLC6A3 / DAT1***	rs28363170	0.78	0.13	**0.05**
***BDNF***	rs6265	0.52	0.33	0.78

**Table 2:** R squared (marginal and conditional, i.e., for fixed effects only and combined fixed effects and random intercept) and Akaike Information Criteria (AIC) for the models with and without Exercise (YES/NO), time (post-motor practice and seven-day retention) and the identified SNPs. Smaller AIC values reflect a better goodness of fit. AIC values are derived from models fit using maximum likelihood (ML). A larger marginal R-squared (R^2^(m)) reflects a higher proportion of accounted variance from the fixed factors alone, whereas a larger conditional R-squared (R^2^(c)) indicates a higher proportion of variance explained by both fixed and random factors.

Fitted Models	AIC	LMM R^2^(m)	LMM R^2^(c)
**~ Time × SNP_1_ + Time × SNP_2_ + … + …**	728	0.04	0.80
**~ Exercise × Time**	717	0.10	0.78
**~ Exercise × Time × SNP_1_ + Exercise × Time × SNP_2_ + … + …**	720	0.19	0.84

## Appendix A

### Appendix A.1. Participants

This retrospective analysis includes data from participants in two previous studies (*n* = 52) published from this group [14,15] as well as additional data from 13 participants (Control (CON) = 7, immediate high intensity exercise (EXE90) = 6) participating in an equivalent pilot study. This meant that 65 neurologically intact, able-bodied, right-handed males (24.3 ± 2.5 years) were included in the analysis (Table A1). Right-handedness for each participant was evaluated with the Edinburgh Handedness Inventory (89.0 ± 18.1) [67]. At the time of enrolment in the study, all the participants were naïve to the visuomotor accuracy task (VAT) used to investigate motor skill learning and procedural memory. All the participants gave their written informed consent prior to testing. The experiments were approved by the local ethics committee for the Greater Copenhagen area (protocol H-2-2011-032), and the study was performed in accordance with the declaration of Helsinki.

**Table A1:** Characteristics of study participants, baseline and post-acquisition motor performance (VAT score, mean ± SD). BMI = Body Mass Index, VO_2peak_ = Maximal relative oxygen uptake, VAT = visuomotor accuracy tracking task.

Group	Control	Exercise			
		EXE90	EXE45	EXE90 + 1 h	EXE90 + 2 h
**Number of participants**	16	14	11	12	12
**Age (years)**	24.4 ± 2.8	25.4 ± 2.7	23.6 ± 2.4	24.1 ± 2.3	23.6 ± 2.0
**Weight (kg)**	78.33 ± 8.3	78.9 ± 11.1	80.6 ± 6.7	80.4 ± 6.7	78.8 ± 13.1
**Height (cm)**	185 ± 7.0	182 ± 5	187 ± 7	184 ± 8	182 ± 7
**BMI (kg/m^2^)**	22.9 ± 2.0	23.8 ± 2.5	23.2 ± 1.3	23.8 ± 1.9	23.6 ± 2.8
**VO_2peak_ (mL O_2_· kg^−1^·min^−1^)**	51.99 ± 4.0	49.5 ± 7.4	49.6 ± 3.9	49.0 ± 5.6	50.4 ± 6.9
**Baseline motor performance (VAT)**	44.6 ± 11.6	43.8 ± 12.4	51.3 ± 9.6	52.5 ± 8.8	50.9 ± 7.5
**Post-acquisition motor performance (VAT)**	68.1 ± 6.3	68.6 ± 5.2	71.9 ± 4.3	72.8 ± 4.8	71.9 ± 4.8

### Appendix A.2. Study Design

The study involved four visits to the lab for each participant. After giving written informed consent and filling out questionnaires related to amount and quality of sleep as well as physical activity and handedness, a full-blood sample for genotyping was drawn from each of the participants. Blood samples were coded and immediately stored in a minus 60 °C freezer for later analysis (see below). The second visit (i.e., the main experiment, one to seven days later) consisted of familiarization and motor practice on the VAT, whereas the third and fourth visits consisted of 24-h and 7-day retention tests. The study design is outlined in Figure 1. Allocation of the participants to intervention groups was stratified for baseline performance, age, VO_2max_, and performance on two cognitive tests of spatial working memory and sustained attention, respectively. Participants were allocated to one of five experimental groups: a resting control group (CON), a low-intensity group (EXE45) and a high-intensity group (EXE90) both engaging in aerobic exercise 20 min post-motor skill acquisition, along with a high-intensity group engaging in aerobic exercise one hour post-motor skill acquisition (EXE90 + 1 h), and a high-intensity group engaging in aerobic exercise two hours (EXE90 + 2 h) post-motor skill acquisition. The 13 participants in the pilot experiments preceding the previously published work were randomly allocated to the EXE90 or CON groups.

### Appendix A.3. Graded Maximal Exercise Test

Participants’ aerobic fitness was assessed via a graded maximal exercise test, and blood lactate samples were collected at increasing workloads. The test was conducted following the protocol used in previous studies by this group [16,17,21]. Peak oxygen consumption was determined when at least one of the following criteria was met: a plateau in the VO_2_ curve, a respiratory exchange ratio ≥ 1.1, an inability to maintain 80 revolutions per minute and/or volitional exhaustion. Mean values for relative VO_2peak_ and peak power output (W_max_) for each group can be seen in Table A1.

### Appendix A.4. Visuomotor Accuracy Tracking Task (VAT)

A standardized introduction and familiarization to the VAT was conducted for all the participants. A schematic overview of the VAT setup can be seen in Figure 1. Each VAT trial consisted of a fixed target consisting of a modified triple sine wave curve presented on a computer screen. Participants were required to track the target as accurately as possible by moving a cursor trace up and down, with wrist extension force moving the cursor upwards and flexion force moving it downwards. Following each trial, augmented feedback on performance was presented as a numerical motor performance score, and the participant’s trace was presented with the target trace. The numerical score range was 0 to 100, with 100 representing a perfect trace of the target. Augmented feedback was only presented during motor skill acquisition, not during delayed retention tests [68]. Trials were separated by a one-second pause. The VAT was performed on three occasions: at the main experiment (acquisition) and at the one-day and seven-day retention tests. The acquisition phase consisted of five separate blocks of 20 trials (100 trials in total), with each block taking four minutes to complete. Blocks of motor practice were interspersed by two-minute breaks, giving a total time of 28 min for the acquisition session. The retention tests at one and seven days also consisted of one block of 20 trials. Mean performance in trials two to 20 in block one was taken as the baseline motor performance, while the mean performance in trials two to 20 in block five represented post-acquisition motor performance, and the mean performance in trials two to 20 seven days later represented long-term retention. A total of 13 participants (CON = 7 and EXE90 = 6) performed seven blocks consisting of three sets of eight different targets (in randomized order, see [16] for details). Behavioral data from these 13 participants were pooled with data from the previously reported studies to increase statistical power. The retention tests consisted of one practice block consisting of two sets of eight different targets for this group. Changes in performance from the last block of motor practice to the seven-day retention test were used as an operationalized measure of long-term retention. This interval was chosen based on the results from the previous publications, where a larger effect of exercise were reported seven days after compared to 24 h after practice ended. It is accordingly likely that this measure is more sensitive to interactions between exercise, genotypes, and consolidation processes.

### Appendix A.5. Exercise Protocol

The exercise protocol has been described previously by Thomas et al. [14,15]. Total exercise time was limited to 17 min in order to avoid fatigue and/or dehydration, which could potentially have a negative effect on psychomotor and cognitive performance [69,70,71]. Participants warmed up for two minutes on a cycle ergometer (Ergomedic 939E, Monark, Sweden) at 50 W for the EX45 group and at 100 W for the remaining groups followed by three blocks of cycling at 90% or 45% (EX45) of W_max_, respectively. Participants were required to keep a cadence of ≥80 RPM during both the two-minute lower intensity active interval (60% or 25% of W_max_) as well as the higher intensity work intervals. Heart rate (Polar Electro, Kempele, Finland) and the rating of perceived exertion (RPE) values (Borg Scale) [72] were recorded during all the intervals, and measures of blood lactate (Accutrend^®^ Plus System, Roche Diagnostics, Rotkreuz, Switzerland) were taken at rest prior to exercise, at the completion of each work interval (one, two, and three) and then again at five minutes post-exercise completion.

### Appendix A.6. DNA Purification and Preparation

A total of 65 blood samples were obtained. The DNA was purified from 200 mL of full blood using the QIAamp DNA blood mini kit (Qiagen, Hilden, Germany) as recommended by the manufacturer.

Samples were genotyped using the iPLEX^®^ Gold kit (Agena Biosciences, Inc., Hamburg, Germany). The PCR contained: 1.1 µL of H_2_O, 0.5 µL of PCR buffer, 0.8 µL of MgCl_2_ (25 mM), 0.1 µL of deoxynucleotide mix (25 mM), 1.3 µL of forward/reverse primer mix (0.5 µM each), 0.2 µL of Hot Star Taq (5 U/µL), and 2 µL of DNA (10 ng/µL) per sample. All the primers are shown in Table A2. The PCR was performed in a GeneAmp PCR system 9700 thermal cycler (Thermo Fisher Scientific, Waltham, MA, USA) with the following conditions: denaturation at 94 °C for 2 min followed by 44 cycles of 94 °C for 20 s, 62 °C for 30 s, and 72 °C for 1 min, followed by 72 °C for 3 min.

The PCR products were treated with a cocktail of 1.53 µL of H2O, 0.17 µL of Test Solution buffer, and 0.3 mL of shrimp alkaline phosphatase (Agena Biosciences, Inc., Hamburg, Germany) per sample. The following PCR reaction was performed in a GeneAmp PCR system 9700 thermal cycler (Thermo Fisher Scientific, Waltham, MA, USA) at 37 °C for 30 min followed by 75 °C for 15 min.

The single-base extension reaction contained 0.619 µL of H_2_O, 0.2 µL) of iPLEX buffer, 0.2 µL of iPLEX pro Termination mix, 0.94 µL of primer mix (0.74–1.46 mM, Agena Biosciences, Inc., Hamburg, Germany), and 0.041 µL of iPLEX1-enzyme per sample. The SBE reaction was performed on a GeneAmp PCR system 9700 thermal cycler (LT-AB) with the following conditions: denaturation at 94 °C for 30 s followed by five cycles of 94 °C for 5 s, followed by 52 °C for 5 s, followed by 40 cycles of 94 °C for 5 s, 52 °C for 5 s, 80 °C for 5 s, followed by 72 °C for 3 min.

A total of 40 µL of molecular grade water and ion exchange resin (Agena Biosciences, Inc., Hamburg, Germany) was added to each sample. Samples were rotated for approximately 5 min on a tube rotator (VWR) and centrifuged at 3600 rpm for 5 min. All the SBE products were spotted twice on the SpectroCHIP array (Agena Biosciences, Inc., Hamburg, Germany) using the RS1000 nanospotter (Agena Biosciences, Inc., Hamburg, Germany). Results were visualized on the MassARRAY analyzer 4 system (Agena Biosciences, Inc., Hamburg, Germany) using the autorun settings. All the samples were typed in duplicate. All the analyses were performed with the results from the first spot from each sample.

### Appendix A.7. Genotype Data Analysis

The MassArray TYPER 4.0 genotyping software analyzed the results in real time using a Gaussian mixture model for cluster analyses. The credibility of the SNP calls were evaluated as a posterior probability using a non-disclosed formula in the MassArray TYPER 4.0.20 software (Agena Bioscience, Inc., Hamburg, Germany), and the SNP calls were divided into three groups: ancestral, minor allele, or both genotype calls. The groups were rated as 0 = ancestral, 1 = both, or 2 = minor allele. The low probability SNP calls were not accepted as genuine SNP genotypes by the TYPER 4.0 genotyping software. If no extended SBE primers were detected at a locus, the genotype call was categorized as ‘no alleles’. The primer sequences can be found in Table A2.

**Table A2:** Primer sequences.

Gene ID	SNP ID	Forward Primer Sequence	Reverse Primer Sequence
***COMT***	rs4680	ACGTTGGATGACCATCGAGATCAACCCCG	ACGTTGGATGTTTTCCAGGTCTGACAACGG
***DRD2***	rs6277	ACGTTGGATGCATTCTTCTCTGGTTTGGCG	ACGTTGGATGACCAGCTGACTCTCCCCGA
***DRD3***	rs6280	ACGTTGGATGCATAGTAGGCATGTGGGCG	ACGTTGGATGCTCTGGGCTATGGCATCTCT
***DRD2***	rs1076560	ACGTTGGATGTAAAGCCGGACAAGTTCCCA	ACGTTGGATGTGTGGTGTTTGCAGGAGTCT
***DRD1***	rs686	ACGTTGGATGAGAGTCTCACCGTACCTTAG	ACGTTGGATGCCTGAACTCGCAGATGAATC
***BDNF***	rs6265	ACGTTGGATGTTGTTTTCTTCATTGGGCCG	ACGTTGGATGGCTTGACATCATTGGCTGAC
***ANKK1***	rs1800497	ACGTTGGATGTCAAGGGCAACACAGCCATC	ACGTTGGATGGACATGATGCCCTGCTTTCG
***PPP1R1B***	rs907094	ACGTTGGATGTGAAGGTCATCAGGCAGTCT	ACGTTGGATGGGACGTCCTCGTATACTCAA

In accordance with previous reports [32,73,74,75,76] each SNP was converted to binary variables by collapsing minor allele carriers (minor allele homozygotes and heterozygotes) with the secondary aim to even out genotype distribution (see Table A3 for Allele distribution).

### Appendix A.8. Statistical Analysis

The effects of the nine physiologically relevant polymorphisms were analyzed individually using linear mixed effect models (LMM) with SNP (two levels), exercise (two levels; YES/NO), and time (two levels; post-motor learning and seven-day retention) as independent variables (Table 1); VAT performance (total time on target) was fitted using the *lme4* R-package [77]. ‘Participants’ were added as random intercepts. Using the properties of the R-package *lmerTest* [78], F-values and p-values were computed for the individual LMMs. Assuming an alpha value of 0.05, three loci were found to interact with exercise and time. In addition, one SNP showed a significant effect with time (SNP × time), and a strong tendency toward a significant interaction with both exercise and time (exercise × time × SNP) (*p* < 0.10). Planned contrasts were computed to visualize and compare the time-dependent impact of SNPs and exercise independently and in combination using the *multcomp* functionalities [79]. These comparisons were adjusted using the single-step method. Additionally, the identified SNPs were added to a multivariate LMM fitted with VAT performance as the dependent variable, and the interactions between exercise and time alongside the four SNPs as the independent variables (exercise × time × SNP) to evaluate the independent and combined effects. Next, in concordance with previous reports addressing the effects of genotypic variation on motor skill learning [31], relative goodness-of-fit was evaluated by means of the Akaike Information Criteria (AIC) that estimates model parsimony penalized for the inclusion of variables for linear mixed models. In this study, these included exercise × time and/or exercise × time × SNP interaction terms on datasets of complete cases using a maximum likelihood fitting approach. Furthermore, marginal and conditional R-squared (R^2^(m) and R^2^(c), respectively) values were extracted as an index of the amount of variance accounted for by the fixed effects alone, and the fixed and random intercepts of the LMM models combined, respectively [80]. This was done using the *MuMIn* R-package [81].

**Table A3:** Allele distribution across participants in the different intervention groups.

SNP ID	rs1076560		rs1800497		rs907094		rs28363170	
Allelic combination	*C*/*C*	*C*/*A* + *A*/*A*	*C*/*C*	*C*/*T* + *T/T*	*T*/*T*	*C*/*T* + *CC*	*10*/*10*	*10*/*9* + *9*/*9*
CON	11	5	10	4	13	3	8	8
EX90	13	1	13	1	5	8	7	6
EX45	7	4	7	4	7	3	5	5
EX90 + 1 h	11	1	9	2	7	5	7	7
EX90 + 2 h	11	1	10	2	8	4	5	7
Not Identified	0	*N*/*A*	3	*N*/*A*	2	*N*/*A*	0	*N*/*A*
